# Mr. Paget’s Report Continued

**Published:** 1844-03-09

**Authors:** 


### RECORD OF MEDICAL SCIENCE.

### MR. PAGET'S REPORT CONTINUED.

#### CIRCULATION.

Dr. Marshall Hall, in a paper on the 'Circulation in the Acardiac Fœtus,' has given proof that the pulsatory movement of the blood may, under certain circumstances, be communicated to the blood in a second set of capillaries. He placed the pectoral fin of an eel in the field of a microscope, and compressed it by the weight of a heavy probe. The movements of the blood in the capillaries became obviously pulsatory, their pulsations being synchronous with the contractions of the ventricle. He adduces this fact in support of the probability that the circulation in the acardiac fœtus is maintained by the force of the heart in the perfect twin fœtus, by which the blood is driven through the capillaries of the placenta into the umbilical vein of the acardiac fœtus, and thence through its venous capillaries into the aorta, and along the umbilical arteries to the placenta again. And the fact is equally important as an additional evidence of the general propagation of the force of the heart through one or even two sets of capillary vessels.

M. Poiseuille's observation of the influence of cold on the capillary circulation is mentioned in the last Report, (p. 44.) He has further shown that the influence of some other agents is similar in organic and in inorganic capillary tubes. By adding successively acetate of ammonia, nitrate of potash, and alcohol to the blood, he found that the first two accelerated, and the last retarded its flow. They produced the same effects when added to serum which was made to pass through inorganic capillary tubes; as, indeed, might be expected, seeing that in both cases the bulk of the fluid moves, not upon the walls of the tube, but upon the layer of fluid which adheres and remains at rest upon the walls. Applying these results to determine the rate at which blood passes from one jugular vein to the other through the lungs, heart, &c., he found that the passage was made, in horses, in from eighteen to twenty-four seconds, when acetate of ammonia or nitrate of potash was added to the blood, but in from forty to forty-five seconds when alcohol was added.

According to Mr. T. Wharton Jones, the congestion which succeeds to the temporary acceleration of the capillary circulation in an inflamed part, is due to the red-blood corpuscles adhering together (in the manner already described,) and to the walls of the vessels till stagnation occurs; and he has shown that the same arrest of the blood takes place when capillaries are touched with a solution of salt, or when a stream of carbonic acid is directed against those of the frog's lung. From these last facts he suggests with much probability that the stoppage of the circulation in the capillaries when certain salts are added to the blood, and that which takes place in asphyxia, depend on a similar adhesion of the corpuscles. With regard to asphyxia, his observations agree in their tendency with those of Dr. John Reid on the stagnation of the blood, independent of any adherent mechanical hindrance, when nitrogen is inhaled; and the action of carbonic acid in making the corpuscles cohere and assume the most favorable condition for the formation of a buffy coat, gives additional probability to the observations already quoted from Dr. Polli.

#### RESPIRATION.

*Respiratory Movements.* MM. Beau and Maissiat, have published some investigations in the physiology of respiration. Revising the forgotten opinions of Haller and Boerhaave, they have pointed out the very different characters of the respiratory movements in men, women, and children. They distinguish three types of these movements. 1. The *abdominal;* in which the visible movements are entirely in the abdominal walls, and especially in their anterior part, the ribs being unmoved, except when the body rests on the side. 2. The *inferior costal;* in which the movement takes place chiefly in the lower ribs, from the seventh inclusive downwards; those above the seventh moving very little, and the less, the higher they stand; and the lower end of the sternum ascending, though in a less degree than the ribs expand. 3. The *superior costal;* in which the movement is effected chiefly in the upper ribs, (especially the first,) which are carried upwards and outwards, and carry with them the clavicles and sternum.

In infants, and often to the third year of life, the respiration is of the abdominal type in both sexes. After the third year, the superior costal type is generally observed in girls, and the inferior costal in boys, and after puberty, the difference becomes more striking. Nearly all women breathe with the upper half of the chest, and nearly all men with the lower half and the abdomen. The mode of respiration in women has no connexion with their wearing of stays, but is probably adapted to the little capacity for breathing with the lower part of the chest during pregnancy. The difference is maintained, in general, even in dyspnœa; only, when it is extreme, a person whose natural respiration is according to any one of these types, may exhibit combinations of the movements proper to the others.

The quiet respiration of the rabbit and the cat is abdominal; their excited respiration is abdominal and inferior costal; that of the dog is always inferior costal; that of the horse is abdominal, except in sighing or when *blown*, when it becomes inferior costal, like that of man. These animals were used in experiments in which many of the actions of the respiratory muscles were observed.

On the anatomy of the osseous parts of the respiratory organs, the authors point out that the intercostal spaces are always proportionately widest between those ribs which are most moved in respiration; the superior are the wider in women, the inferior in men. In men, too, there is a remarkable distance between the sixth and seventh ribs, and the seventh and three following it often form a great projection. The articulations of the last two ribs with the spine are very lax, and their anterior ends being free, they follow the movements of the abdominal walls in which they are imbedded; they commonly descend in abdominal inspiration, and ascend in the inferior costal movement. The first rib is peculiarly moveable in women, and those who breathe like them; nearly, or quite immoveable in men and animals which breathe habitually with the lower ribs and abdomen. And herein is the solution of the question of the mobility or immobility of the first rib, as well as of that respecting the relative degrees of freedom of motion in the other ribs; they vary according to the peculiar type of the respiratory movements.

The shortness and early ossification and anchylosis of the first costal cartilage, make the sternum participate much more in the movements of the upper ribs than it does in those of the lower ones; hence, the antero-posterior enlargement of the chest in inspiration is much greater in women than in men. The increase of the intercostal spaces in inspiration is directly proportionate to their natural width; greatest, therefore, above in women, and below in men. In both, the increase is far greater anteriorly than it is posteriorly. In forcible expiration, the width of the intercostal spaces may be reduced to considerably less than it is in ordinary expiration.

MM. Beau and Maissiat investigated also at great length the actions of the respiratory muscles, both by feeling and looking at them while in action, and by vivisections of dogs. Their conclusions, so far as the muscles are concerned in respiration, are briefly as follows, and many of them may be confirmed by observation on one's own person. *Intercostals:* In inspiration, they are elongated, and become hard and concave on their outer surface; in quiet expiration, they are moderately shortened, and become less hard and flat; in complex and forcible expiration, they become prominent and very short and hard. They are therefore muscles for forcible expiration, like their analogues, the oblique muscles of the abdomen; their hardness in inspiration is due to their being stretched; but their contraction (except by their elasticity) is only seen in forced expirations or in efforts.

[For many reasons, this conclusion must be considered very doubtful. The experiment on which the authors chiefly found their belief that the intercostals cannot raise the ribs, consisted in cutting through the pectoral muscles and the whole length of the intercostals between the sixth and seventh ribs on both sides: after this was done the lower ribs were still raised in inspiration (as they suppose) by the diaphragm. Perhaps no conclusion ought to be drawn from the results of such mutilation; but M. Debrou (Gazette Médicale, Jan. 3, 1843,) having repeated the experiment, with the addition of cutting the diaphragm from the ribs, and having found that the ribs were still raised in inspiration, maintains that the five lower ribs are thus raised by their intercostal muscles, and that the sixth, from which the intercostals above were cut away, is *pushed* up by the fifth. The following arguments appear to me conclusive in favour of the usually inspiratory action of the intercostals. 1. When the spinal cord is injured below the origins of the phrenic nerves and above those of the intercostal nerves, the ribs are very nearly motionless in respiration, for the intercostal muscles are paralysed though the diaphragm is active. 2. The upper ribs are chiefly moved in the superior costal respiration, though the diaphragm cannot act upon them. 3. The levatores costarum, which can act in inspiration alone, have an arrangement exactly analogous to that of the external intercostal muscles. 4. Whenever the intercostal muscles are affected by diseases in which the pain is increased by muscular contraction, there is an increase of pain in inspiration.] The authors believe also (and with more probability, for whatever be their ordinary action, the intercostals may, in extraordinary circumstances, act in either direction,) that in forcible expiration they serve to make the whole walls of the chest rigid and resisting, so that they may not be distended by the eccentric impulse of the lungs, which are compressed on every side, and especially by the diaphragm. *Levatores costarum:* supposed (but improbably) to be not concerned in respiration, but to serve for maintaining the spine erect. *Infra costales:* probably muscles for forcible expiration, like the internal intercostals. (?) *Triangularis sterni:* a muscle of expiration, by drawing together the sternum and the costal cartilages. *Scaleni:* muscles of inspiration, especially in the superior costal type of movements, but chiefly flexors of the head. *Sternomastoid:* auxiliary to the scaleni in forcible inspiration. *Trapezius:* its upper border assists in forcible inspiration, its lower border in forcible expiration. *Levator anguli scapulæ:* acts with the upper part of the trapezius in violent inspiration. *Subclavius:* depressor of the clavicle after forcible inspiration. (?) *Latissimus dorsi:* its lower border acts in forcible expiration, as one may find by feeling the posterior wall of the axilla while coughing; at the same time it makes rigid those parts of the walls of the chest and abdomen on which it lies, and it presses in the lower ribs. *Serratus magnus:* acts in forcible inspiration, but chiefly (as was shown in a patient in whom it alone was paralysed,) it serves, by co-operating with the deltoid in raising the arm. *Serratus posticus superior:* not a respiratory muscle, (?) but an extensor of the neck. *Serratus posticus inferior:* expiratory. *Pectoralis major:* its lower quarter is a muscle of inspiration, its upper three-fourths form one of expiration, but it does not act except in dyspnœa. *Pectoralis minor:* its lower half acts habitually (?) as a muscle of inspiration.

As to the action of the diaphragm, the authors believe that it produces, 1. Elongation of the thoracic cavity, especially in the abdominal type of respiration. 2. Increase of the transverse diameter, by elevating and turning outwards the lower ribs, as in the experiment quoted in a preceding note; and this especially in the inferior costal respiration. 3. Occasionally, in infants, the depression of the costal cartilages. The second of these actions of the diaphragm is also described by M. Magendie. The true mode of action is probably this: when the muscular fibres of the diaphragm contract, its central portion descends, and at the same time traction is exercised on the ribs at the peripheral ends of the fibres; and when the resistance to the descent of the diaphragm is greater than the resistance to an upward motion of the ribs, these are raised by the fibres which are attached to them, and whose direction, even in moderate inspiration, is nearly vertical. And this drawing upwards of the ribs is necessarily converted into a movement upwards and outwards by the limited and peculiar mobility of their attachments to the vertebræ and sternum. The third assigned action, that of the occasional depression of the inferior costal cartilages in children, is more reasonably ascribed by Mr. Alexander Shaw to this, the most pliant part of the walls of the chest, being pressed in by the atmosphere when the other parts of the chest are expanded to a size which the lungs cannot attain, on account either of disease of their structure, or of obstruction to the free entrance of air through the larynx and trachea.

*Structure of the Lungs.* Mr. Addison has given an account of the anatomy of the minute air-passages which, while it confirms nearly all that Reisseissen observed, is more complete, and very probably true. In the fœtus the ultimate bronchial subdivisions are tubular; they have a regularly branched arrangement, ramifying symmetrically in all directions, and terminating without anastomoses in closed extremities which are generally situated at the boundaries of the lobules. But when an animal has respired, the entrance of the air into the lungs distends the lobules, and the ultimate bronchial subdivisions undergo a great change. The membrane composing each of them offers only a feeble resistance to the pressure of the air, and is pushed forwards and distended laterally into rounded inflations, forming a series of communicating cells, which meeting on all sides those of the adjoining bronchial subdivisions, are moulded by the mutual pressure into various hexagonal and pentagonal forms. These distended passages (something like large beaded tubes) Mr. Addison calls *lobular passages;* and a section of them shows the *oval foramina* leading from cell to cell, which are so conspicuous in a thin layer of inflated and dried lung. The *air cells,* according to this account, are the inflated parts of the *intralobular* bronchial subdivisions; and those of each lobule form a distinct system, having no communication with those of the adjacent lobules, except in the common trunk from which the intralobular bronchi of each system are derived. The air-cells are from 1-200 to 1-500 of an inch in diameter; and the oval foramina are from 1-60 to 1-150 of an inch or less in diameter. The blood-vessels lie upon each *lobular passage,* and between each two of them.

*Capacity of breathing.* M. Bourgery's examinations of the structure of the lungs are detailed in vol. XIV, p. 546. They may easily be reconciled with the more probable account of Mr. Addison, from which they chiefly differ in that the minutest branches of the bronchi are described in them as freely anastomosing, so as to form a series of labyrinthic canals; and that the constrictions of the tubes by which they are formed into cells or loculi are said to be due to annular vessels surrounding them. M. Bourgery has more recently examined the relations of the varying structure of the lungs in different ages and sexes to their functional capacity. The subjects examined were fifty males and twenty females, and the deductions are as follows: 1. The measure of respiration (that is, I think, the proportion between the quantity of air which can be taken in by a forced inspiration, and the quantity which the lungs just previously contained) is always the greater the more youthful and lean the person is: strength and health do not in this regard compensate for youth. 2. The measure in males is twice as great as in females of the same age. 3. The function is at its highest point in both sexes at thirty years of age—the age which corresponds with the completest developement of the aërial capillary plexus, or finest branches of the bronchi. At this age a forced inspiration increases the air in the chest from 2·5 to 4·3 litres in males, and from 1·1 to 2·2 in females. The boy of fifteen inspires two litres, the man of eighty, 1·35. 3. The volume of air necessary for an *ordinary* inspiration increases with advancing age; and this increase exactly represents the diminution of the energy of the pulmonary hematosis. 4. The capacity of the lungs for forcible inspiration increases from infancy to the age of thirty, doubling itself in twenty-three years. After thirty it diminishes one fifth in the first twenty years; one fifth more in the next ten; and nealry one half in the next twenty; and this gradual decrease of capacity for forcible inspiration is true of all persons, although one may have a greater general capacity of respiration than another of the same age. Hence the young person possesses a great capacity of respiration, as it were, in reserve; the old man has little, and is therefore unfit for great exertion.

*Exhalation of carbonic acid.* MM. Andral and Gavarret state the following as the results of experiments made in sixty-two persons (thirty-six males and twenty-six females,) to determine the quantity of carbonic acid exhaled in breathing: 1. At all ages beyond eight years the exhalation is greater in males than in females. 2. In males it regularly increases in quantity from eight to thirty years of age; from thirty to forty it is stationary or diminishes a little; from forty to fifty the diminution is greater; and from fifty to extreme age it goes on diminishing till it scarcely exceeds the quantity at ten years. 3. The quantity of carbon exhaled in the form of carbonic acid in one hour by males of different ages is as follows;—at eight years, 77·5 grains; at fifteen, 135 grains; at twenty, 176·7 grains; between thirty and forty, 189 grains; between forty and sixty 156 grains; between sixty and eighty, 142·5 grains; and in a man 102 it was only 91·5 grains. 4. In females the same proportionate increase goes on to the time of puberty when the quantity abruptly ceases to increase, and remains stationary so long as they continue to menstruate. When, however, menstruation has ceased, the exhalation of carbonic acid begins again to augment; and, then again, in advancing years, decreases as it does in men. Thus before puberty the quantity of carbon exhaled by girls in an hour is ninety-nine grains; aud so it continues while the habit of menstruation continues; afterwards, from thirty-eight to forty-nine years of age, it increases to 130 grains; from fifty to sixty again falls to 113 grains; from sixty to eighty is reduced to 105 grains; and in a woman of eighty-two, was only ninety-three grains. 5. In amenorrhea the exhalation is always increased. 6. In pregnancy the exhalation is equal to that which is natural soon after the cessation of menstruation. 6. *Cæteris paribus*, the more robust a person is the more carbonic acid is exhaled; but the differences are not great. 7. The maximum of exhalation was in a strong man of twenty-six, who in an hour exhaled carbonic acid containing 218·5 grains of carbon; the proportionate minimum in a weak man of forty-five, who exhaled in the same time only 139·5 grains; 8. The influences of the weights of persons, of the capacities of their chests, and of the extent of the respiratory movements, are not great.

